# Trends and Characteristics of Candidemia in Patients With Suspected Sepsis: A Two-Year Retrospective Study From a Tertiary Hospital in Uttarakhand

**DOI:** 10.7759/cureus.86241

**Published:** 2025-06-17

**Authors:** Rajender Singh, Barnali Kakati, Garima Mittal

**Affiliations:** 1 Microbiology, Himalayan Institute of Medical Sciences, Swami Rama Himalayan University, Dehradun, IND

**Keywords:** antifungal susceptibility test, candidemia, non-albicans candida, sepsis, tertiary care hospital

## Abstract

Introduction

Candidemia is a significant cause of morbidity and mortality in patients with sepsis, particularly in tertiary care settings. Shifts in *Candida* species distribution and antifungal resistance underscore the need for localized epidemiological surveillance.

Aim and objective

This study aimed to evaluate the prevalence, species distribution, clinical risk factors, and antifungal susceptibility patterns of *Candida* isolates in sepsis patients admitted to a tertiary care hospital in Uttarakhand, India.

Methods

A retrospective observational study was conducted from February 2022 to January 2024. Blood culture records of 17,712 sepsis patients were reviewed, among which 312 cases of candidemia were identified. Blood cultures were processed using the BAC-T ALERT system, and isolates were identified to the species level using conventional microbiological methods and the Vitek-2 system. Antifungal susceptibility testing was performed as per Clinical and Laboratory Standards Institute (CLSI) guidelines. Relevant demographic, clinical, and risk factor data were extracted and statistically analyzed using SPSS version 26.0. Associations between *Candida* species and clinical variables were evaluated, with p-values < 0.05 considered statistically significant.

Results

The prevalence of candidemia among sepsis patients was 1.76% (312/17,712), with a mean age of 37.7 years and a male-to-female ratio of 1.72:1. Non-*albicans Candida* species predominated, led by *C. tropicalis* 84 (26.9%), followed by *C. albicans* 68 (21.8%), *C. parapsilosis* 58 (18.6%), and *C. pelliculosa* 37 (11.8%). *C. tropicalis* was significantly associated with chronic kidney disease: 21 (25.0%) (p = 0.00018); *C. parapsilosis* with low-birth-weight neonates: 15 (25.9%) (p = 0.00024); and *C. glabrata* 12 (63.2%) with diabetes mellitus (p = 0.0067). *C. pelliculosa* was predominant in neonates: 31 (83.8%) (p < 0.001). Most isolates were susceptible to fluconazole 284 (91.0%), voriconazole 290 (93.0%), amphotericin B 296 (94.9%), and echinocandins 300 (96.1%). Fluconazole resistance was highest in *C. krusei* 2/3 (66.7%) and *C. auris* 8/8 (100%), while echinocandin resistance was noted in *C. glabrata, *caspofungin 7/19 (36.8%) and micafungin 3/19 (15.8%).

Conclusion

The study highlights an epidemiological shift toward non-*albicans Candida* species, particularly *C. tropicalis* and *C. pelliculosa*, in candidemia cases. Routine species-level identification and antifungal susceptibility testing are crucial for guiding effective therapeutic strategies and supporting institutional antifungal stewardship.

## Introduction

Candidemia, a bloodstream infection caused by *Candida spp.*, poses a serious risk to hospitalized and critically ill patients, with significant associated morbidity and mortality [1]. The increasing prevalence of candidemia has raised considerable concern, particularly in the context of sepsis, where immune compromise, invasive procedures, and prolonged broad-spectrum antibiotic use predispose patients to opportunistic fungal infections [1,2]. Recent studies indicate a shifting epidemiology, with a rise in non-albicans Candida (NAC) species such as *C. tropicalis*, *C. glabrata*, *C. krusei*, and *C. parapsilosis*, which often exhibit varying degrees of antifungal resistance [3,4].

In ICUs, candidemia constitutes a major cause of bloodstream infections, with reported mortality rates ranging from 30% to 40.4% [5,6]. The increasing prevalence of NAC species, many of which demonstrate resistance to fluconazole and other antifungal agents, complicates treatment and underscores the urgent need for enhanced surveillance, early diagnosis, and the implementation of antifungal stewardship programs [7,8]. In the Indian clinical context, *C. tropicalis* has emerged as one of the predominant pathogens causing candidemia, influenced by both environmental and patient-specific factors [9,10].

Patients with sepsis, who frequently have multiple comorbidities and prolonged ICU stays, are at heightened risk of candidemia due to immune dysfunction and the use of invasive devices such as central venous catheters, which facilitate fungal bloodstream invasion [11]. Despite the growing body of evidence on candidemia in critically ill patients, there remains a paucity of detailed, region-specific data regarding the age-wise distribution of *Candida* species, the impact of underlying comorbidities, and antifungal susceptibility profiles in India. This study aims to address these gaps by conducting a comprehensive analysis of candidemia among sepsis patients at a tertiary care hospital in the Sub-Himalayan region, focusing on species distribution, clinical risk factors, and antifungal resistance patterns.

## Materials and methods

Study design

This retrospective observational study was conducted in the Department of Microbiology at Himalayan Hospital, Jolly Grant, Uttarakhand, India. The study spanned a period of two years, from February 2022 to January 2024. Blood samples for fungal culture were obtained from 17,712 patients admitted with suspected sepsis during the study period, encompassing all ICUs (including medical, surgical, and trauma ICUs) as well as general wards across the hospital.

Inclusion criteria

All patients admitted with clinical suspicion of sepsis, from whom blood cultures were obtained for mycological investigation during the study period, were included in the study. The inclusion criteria encompassed all age groups, with no age-based exclusions applied.

Exclusion criteria

Patients with incomplete medical records, previously confirmed fungal bloodstream infections, or inadequate culture samples were excluded from the study.

Ethical approval

The study was approved by the Institutional Ethics Committee (Reg. No. ECR/483/Inst/UK/2013/RR-16), Himalayan Institute of Medical Sciences, Swami Rama Himalayan University, ensuring adherence to ethical guidelines and the protection of patient confidentiality (Approval No. HIMS/RC/2021/204-1, dated 20.09.21).

Sample collection

Blood samples were collected aseptically and processed using the BACT/ALERT automated blood culture system (bioMérieux, France). Positive cultures yielding yeast growth were further analyzed for species-level identification and antifungal susceptibility testing (AFST).

Microbiological diagnosis (confirmed cases)

Identification of Yeast Isolates

Yeast isolates from culture-positive samples were identified to the species level using a combination of conventional microbiological techniques and the VITEK-2 Compact automated identification system (bioMérieux, France).

Antifungal Susceptibility Testing (AFST)

AFST was performed using the VITEK-2 system. Results were interpreted based on Clinical and Laboratory Standards Institute (CLSI) guidelines. Antifungal agents tested included fluconazole, voriconazole, amphotericin B, and echinocandins (caspofungin and micafungin).

Data collection

Sociodemographic and clinical data were retrospectively extracted from electronic medical records. Collected variables included patient age, sex, underlying comorbidities (such as diabetes mellitus and chronic kidney disease), and prior antifungal therapy.

Data analysis

Statistical analysis was conducted using IBM SPSS Statistics for Windows, Version 26.0 (IBM Corp., Armonk, NY). Categorical variables were analyzed using the chi-square test. A p-value of <0.05 was considered statistically significant.

## Results

Table 1 outlines the clinical demographics of suspected sepsis patients diagnosed with candidemia. The overall prevalence of candidemia was 312 (1.76%) among 17,712 suspected sepsis cases. The mean age of affected individuals was 37.7 years, with a male-to-female ratio of 1.72.

**Table 1:** Clinico-demographic characteristics of suspected sepsis patients with candidemia (N = 312).

Parameter	Frequency (%)
Prevalence of candidemia	312 / 17,712 (1.76%)
Mean age (years)	37.7
Male:female ratio	1.72:1

Species-specific distribution patterns were observed across key clinical risk factors. *Candida tropicalis* was significantly associated with chronic kidney disease, 21 cases (25.0%) (p = 0.00018), and hypertension, also 21 cases (25.0%) (p = 0.0074), among 84 cases. *C. parapsilosis* was more frequently isolated in patients with malignancy, 12 cases (20.7%) (p = 0.0004), and in low or very low birth weight neonates, 15 cases (25.9%) (p = 0.00024), among 58 cases. *C. glabrata* showed a strong association with diabetes mellitus, 12 cases (63.2%) (p = 0.0067), among 19 cases. Although these trends suggest potential associations between *Candida* species and underlying comorbidities, the retrospective study design and limited subgroup sizes preclude definitive conclusions. Prospective studies with larger cohorts are warranted to validate these associations (Table 2).

**Table 2:** Risk factors associated with different Candida species isolated in candidemia (N = 312). DM: Diabetes mellitus; HTN: Hypertension; CLD: Chronic liver disease; CKD: Chronic kidney disease; LBW/VLBW: Low birth weight/Very low birth weight; TB: Tuberculosis. *Others include *C. utilis*, *C. famata*, *C. krusei*, *C. lusitanae*, *C. auris*, etc. p-value <0.05 is considered statistically significant.

Risk Factor	*C. albicans* (n = 68)	P-value	*C. glabrata* (n = 19)	P-value	*C. tropicalis* (n = 84)	P-value	*C. parapsilosis* (n = 58)	P-value	*C. pelliculosa* (n = 37)	P-value	Others* (n = 46)	P-value
DM	12 (17.6%)	0.188	12 (63.2%)	0.0067	17 (20.2%)	0.719	7 (12.1%)	0.515	0 (0.0%)	0.586	12 (26.1%)	0.224
Malignancy	4 (5.9%)	0.477	1 (5.3%)	1	6 (7.1%)	0.802	12 (20.7%)	0.0004	0 (0.0%)	1	1 (2.2%)	0.488
HTN	6 (8.8%)	1	5 (26.3%)	0.515	21 (25.0%)	0.0074	6 (10.3%)	0.809	2 (5.4%)	0.562	5 (10.9%)	1
CLD	3 (4.4%)	0.355	1 (5.3%)	1	4 (4.8%)	1	2 (3.4%)	1	0 (0.0%)	1	4 (8.7%)	0.169
CKD	1 (1.5%)	0.332	3 (15.8%)	1	21 (25.0%)	0.00018	5 (8.6%)	0.794	0 (0.0%)	1	5 (10.9%)	0.774
LBW/VLBW	6 (8.8%)	0.507	1 (5.3%)	0.708	1 (1.2%)	0.0021	15 (25.9%)	0.00024	6 (16.2%)	0.0108	6 (13.0%)	0.36
TB	6 (8.8%)	0.284	3 (15.8%)	1	11 (13.1%)	0.214	1 (1.7%)	0.137	2 (5.4%)	0.395	6 (13.0%)	0.187

Table 3 presents the age-wise distribution of *Candida* species isolated from blood cultures. *Candida pelliculosa* was predominantly isolated in neonates, 31 cases (83.8%) among 37 cases (p < 0.001), followed by *C. parapsilosis*, 21 cases (36.2%) among 58 (p = 0.00748), and *C. albicans*, 12 cases (17.6%) among 68 (p = 0.0266), within the same age group. Among adults (18-65 years), the most frequently isolated species were *C. albicans*, 37 cases (54.4%) among 68 (p = 0.0266), *C. tropicalis*, 32 cases (38.1%) among 84 (p = 0.00261), and *C. glabrata*, 16 cases (84.2%) among 19 (p = 0.00186). In children aged 1-18 years, notable species included *C. tropicalis*, 12 cases (14.3%) among 84, and *C. parapsilosis*, 11 cases (19.0%) among 58. These findings demonstrate statistically significant age-specific distribution patterns of candidemia, with certain *Candida* species showing clear predominance in both neonatal and adult populations.

**Table 3:** Age-wise distribution of yeast and yeast-like species isolated from blood cultures. Others include *C. utilis*, *C. famata*, *C. krusei*, *C. lusitaniae*, *C. auris*, etc. p-value < 0.05 is considered statistically significant.

Age Group	*Candida tropicalis* n (%)	*Candida albicans* n (%)	*Candida parapsilosis* n (%)	*Candida pelliculosa* n (%)	*Candida glabrata* n (%)	Others n (%)
Neonate (0-1 month)	12 (14.3%)	12 (17.6%)	21 (36.2%)	31 (83.8%)	2 (10.5%)	8 (17.4%)
Infant (1 mo-1 year)	9 (10.7%)	7 (10.3%)	6 (10.3%)	4 (10.8%)	0 (0.0%)	1 (2.2%)
Children (1-18 years)	12 (14.3%)	3 (4.4%)	11 (19.0%)	0 (0.0%)	0 (0.0%)	6 (13.0%)
Adults (18-65 years)	32 (38.1%)	37 (54.4%)	12 (20.7%)	0 (0.0%)	16 (84.2%)	27 (58.7%)
Elderly (>65 years)	19 (22.6%)	9 (13.2%)	8 (13.8%)	2 (5.4%)	1 (5.3%)	4 (8.7%)
Total	84	68	58	37	19	46
p-value	0.00261	0.0266	0.00748	<0.001	0.00186	0.0272

**Figure 1:**
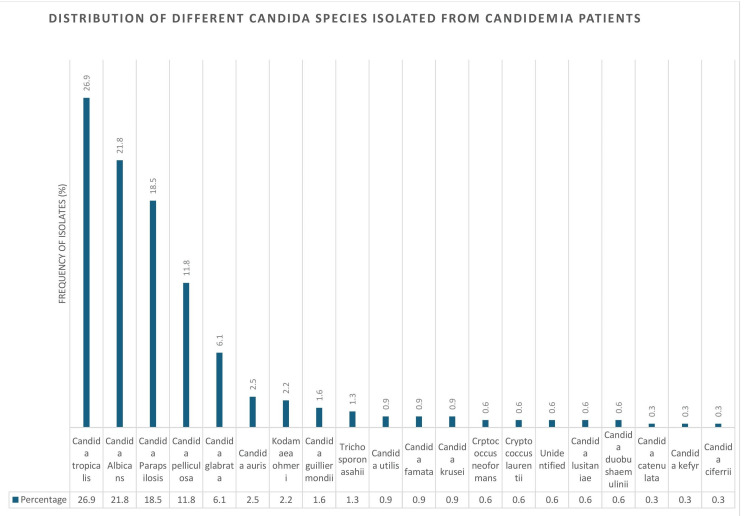
Overall distribution of different Candida species isolated from candidemia patients (n = 312).

Figure 1 shows the distribution of *Candida* species isolated from 312 candidemia patients. *Candida tropicalis* was identified in 84 (26.9%) cases, *C. albicans* in 68 (21.8%), and *C. parapsilosis* in 58 (18.5%). *C. pelliculosa* and *C. glabrata* were isolated in 37 (11.8%) and 19 (6.1%) cases, respectively. Less common species included *C. auris*, 8 cases (2.5%), Kodamaea ohmeri, 7 (2.2%), and C. guilliermondii, 5 (1.6%). Rare species such as *C. ciferri*, *C. kefyr*, and *C. catenulata* were each identified in 1 case (0.3%). These findings demonstrate the predominance of *C. tropicalis* and *C. albicans* in candidemia, along with notable frequencies of *C. parapsilosis* and *C. pelliculosa*.

**Figure 2:**
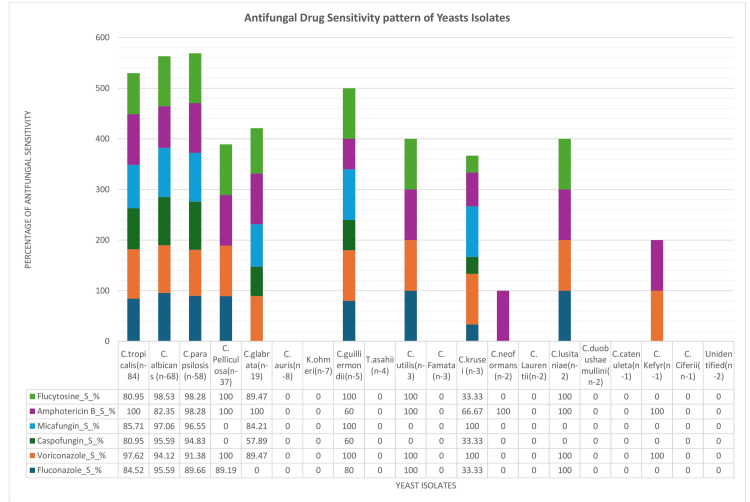
Antifungal drug sensitivity patterns of yeast isolates from blood cultures in candidemia patients (n = 312).

Figure 2 illustrates the MIC-based antifungal susceptibility pattern of 312 yeast isolates from blood cultures, assessed using the Vitek-2 system. *C. tropicalis* showed fluconazole susceptibility in 71 (84.5%) of 84 isolates, *C. albicans* in 65 (95.5%) of 68 isolates, and *C. parapsilosis* in 52 (89.6%) of 58 isolates. Voriconazole susceptibility was observed in over 91% of isolates across these species, while amphotericin B showed susceptibility in more than 82%. Echinocandins and flucytosine demonstrated greater than 90% activity against the majority of common species. Conversely, rare yeasts including *C. auris* (0/8), K. ohmeri (0/7), and *C. krusei* (0/3) exhibited no susceptibility across agents. However, this likely reflects limitations of the Vitek-2 system due to unvalidated breakpoints for these species rather than true resistance. These findings underscore the importance of cautious interpretation and the need for confirmatory antifungal susceptibility testing for rare yeast species when clinically indicated.

**Figure 3:**
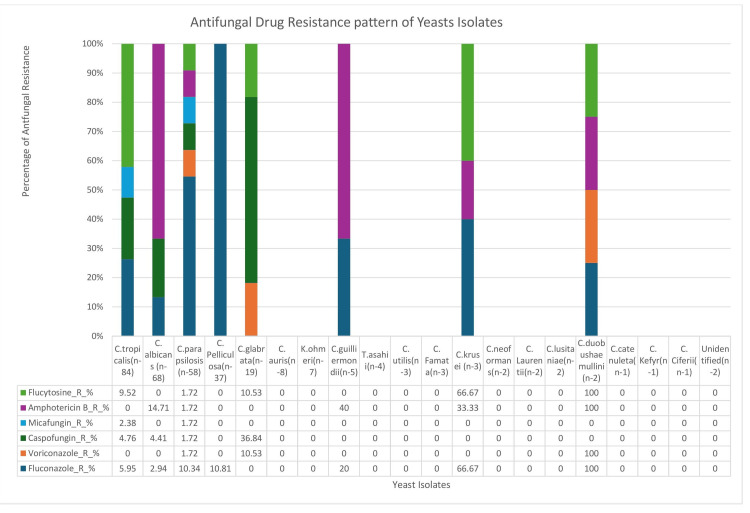
Antifungal drug resistance patterns of yeast isolates from blood cultures in candidemia patients (n = 312).

Figure 3 presents antifungal resistance patterns among 312 yeast isolates from blood cultures using Vitek-2. Fluconazole resistance was low in *C. tropicalis* (5/84; 5.95%), *C. albicans* (2/68; 2.94%), and *C. parapsilosis* (6/58; 10.34%), but was high in *C. krusei* (2/3; 66.67%) and *C. duobushaemulinii* (2/2; 100%). Echinocandin resistance was primarily observed in *C. glabrata*, with resistance to caspofungin in 7 (36.84%) of 19 isolates and micafungin in 3 (15.8%) of 19. Flucytosine resistance was moderate in *C. krusei* (2/3; 66.67%) and *C. guilliermondii* (2/5; 40%). Amphotericin B resistance was generally low but present in *C. albicans* (10/68; 14.71%) and *C. krusei* (1/3; 33.33%). As with susceptibility testing, rare species demonstrated 0% susceptibility, likely due to Vitek-2 system limitations rather than actual resistance, reinforcing the need for confirmatory testing in such cases.

## Discussion

This study provides a comprehensive analysis of candidemia in sepsis patients, with findings consistent with both global and regional trends. The prevalence of candidemia in our cohort was 1.76%, aligning with reports from other ICU settings in India and abroad, where candidemia rates among sepsis patients range from 0.65% to 6.9% [12,13]. The proportion of *Candida tropicalis* isolates (26.9%) observed in the present study is consistent with earlier findings from India, where *C. tropicalis* has been reported as the predominant species in candidemia cases, accounting for up to 43.2% of isolates [7]. In contrast, other reports identify *Candida albicans* as the dominant species [14]. This shift in species dominance may reflect local environmental factors, hospital practices, fungal ecology, and patient demographics, such as a higher prevalence of comorbidities like chronic kidney disease (CKD) and diabetes mellitus, which predispose patients to candidemia [15].

The observed predominance of *Candida pelliculosa* in neonates is noteworthy, as this species has been associated with outbreaks in NICUs due to its ability to form biofilms on medical devices [16]. The high prevalence of *C. albicans* and *C. tropicalis* in adults, particularly those with comorbidities, further highlights the role of immunocompromised states in the pathogenesis of candidemia [17].

Antifungal susceptibility testing revealed that most *Candida* species remain highly susceptible to azoles, in line with previous reports [18]. However, resistance was detected in *Candida krusei* and *Candida auris*, both known for multidrug resistance. The emergence of *C. auris* is particularly concerning due to its environmental persistence and potential to cause healthcare-associated outbreaks [19].

This study underscores the importance of age-specific approaches to candidemia management. Neonates, who were predominantly affected by *C. pelliculosa*, may benefit from enhanced infection control measures and antifungal agents effective against this species. In adults, particularly those with CKD and diabetes mellitus, antifungal stewardship and early initiation of appropriate therapy, guided by susceptibility testing, are critical to improving clinical outcomes.

This study has several limitations. As a retrospective analysis, it is subject to inherent biases, including incomplete or inconsistent clinical documentation. The single-centre design may limit the generalizability of findings to other healthcare settings. Additionally, species-specific associations with clinical risk factors were based on limited subgroup sizes, which may affect statistical robustness.

## Conclusions

Our study highlights the evolving epidemiology of candidemia in sepsis patients, marked by a shift toward non-albicans species such as *C. tropicalis* and *C. pelliculosa*. These findings underscore the need for targeted surveillance and species-specific antifungal therapy guided by susceptibility patterns. Strengthening infection control practices is essential to prevent nosocomial transmission and limit the emergence of resistant strains. Future research should focus on elucidating molecular resistance mechanisms and evaluating novel antifungal agents to address multidrug-resistant *Candida* infections.
